# Targeting hyaluronan metabolism-related molecules associated with resistant tumor-initiating cells potentiates chemotherapy efficacy in lung cancer

**DOI:** 10.1038/s41598-024-66914-0

**Published:** 2024-07-22

**Authors:** Marco Aurelio Díaz, Mariel Fusco, Constanza Arriola Benítez, Fernando Gayet, Ludmila García, Lucia Victoria, Sebastián Jaramillo, Juan Bayo, Mariana Rodríguez Zubieta, Manglio M. Rizzo, Flavia Piccioni, Mariana Malvicini

**Affiliations:** 1 Cancer Immunobiology Laboratory, Instituto de Investigaciones en Medicina Traslacional, Universidad Austral-Consejo Nacional de Investigaciones Científicas y Técnicas, Pilar, Argentina; 2 Servicio de Oncología, Hospital Universitario Austral, Buenos Aires, Argentina; 3 Laboratorio Central, Hospital Universitario Austral, Buenos Aires, Argentina; 4 Programa de Hepatología Experimental y Terapia Génica, Instituto de Investigaciones en Medicina Traslacional, Universidad Austral-Consejo Nacional de Investigaciones Científicas y Tecnicas, Pilar, Argentina; 5 Servicio de Anatomía Patológica, Hospital Universitario Austral, Buenos Aires, Argentina

**Keywords:** Cancer stem cells (CSC), Non-small cell lung cancer (NSCLC), Taxane-chemotherapy, Glycosaminoglycans (GAGs), Hyaluronan (HA), Chemoresistance, Cancer, Molecular biology, Oncology

## Abstract

The success of chemotherapy regimens in patients with non-small cell lung cancer (NSCLC) could be restricted at least in part by cancer stem cells (CSC) niches within the tumor microenvironment (TME). CSC express CD133, CD44, CD47, and SOX2, among other markers and factors. Analysis of public data revealed that high expression of hyaluronan (HA), the main glycosaminoglycan of TME, correlated positively with CSC phenotype and decreased disease-free interval in NSCLC patients. We aimed to cross-validate these findings on human and murine lung cancer cells and observed that CD133 + CSC differentially expressed higher levels of HA, HAS3, ABCC5, SOX2, and CD47 (*p* < 0.01). We modulated HA expression with 4-methylumbelliferone (4Mu) and detected an increase in sensitivity to paclitaxel (Pa). We evaluated the effect of 4Mu + chemotherapy on survival, HA metabolism, and CSC profile. The combination of 4Mu with Pa reduced the clonogenic and tumor-forming ability of CSC. Pa-induced HAS3, ABCC5, SOX2, and CD47 expression was mitigated by 4Mu. Pa + 4Mu combination significantly reduced in vivo tumor growth, enhancing animal survival and restoring the CSC profile in the TME to basal levels. Our results suggest that HA is involved in lung CSC phenotype and chemosensitivity, and its modulation by 4Mu improves treatment efficacy to inhibit tumor progression.

## Introduction

Non-small cell lung cancer (NSCLC) comprises over two-thirds of all cases of lung tumors and remains the leading cause of cancer mortality worldwide^1^. Despite recent evidence revealing the reduction of lung cancer mortality by staging system improvements, such as screening high-risk patients with low-dose computed tomography^2^, most cases of NSCLC are still diagnosed in advanced stages^1,3^. For these patients, surgical resection is not an option. In the absence of EGFR, ALK, ROS1, and BRAF mutations, they are managed with platinum-based doublet chemotherapy^4,5^ combined with immunotherapy (anti-PD-1, anti-PD-L1 or anti-PD-1 + anti-CTLA-4)^6,7^. Although these regimens drove a progressive improvement in median progression-free survival (mPFS), and median overall survival (mOS), some factors still could influence the resistance, or the lack of efficacy observed in patients who experienced disease progression. Conversely, patients with early-stage NSCLC are eligible for surgical resection with curative intent^8,9^; however, a considerable proportion of these patients will experience disease recurrence regardless^3,10,11^.

A key player in explaining these rates of negative clinical outcomes could be a subpopulation of malignant cells within the tumor microenvironment (TME), the lung cancer stem cells (CSC), which possess characteristic functional properties, including self-renewal, resistance to chemotherapeutic agents and to ionising radiation^12,13^, and metastatic potential^14^. CSC also exhibit specific surface markers and transcriptional profiles. Particularly in NSCLC, CSC can be distinguished by CD133, CD44 and Aldehyde Dehydrogenase 1 (ALDH1) expression^15–20^. Potential CSC subpopulations are often identified by the co-expression of described surface proteins as a surrogate measure of their functional properties^21^. Accordingly, expression of putative CSC markers in NSCLC tumor samples has been associated with a higher risk of recurrence^22,23^ and shorter overall survival^24^, indicating that the CSC subpopulation could be a target for more effective antineoplastic therapy.

The TME can modulate cancer cell plasticity toward a CSC phenotype^25,26^ through hyaluronan (HA), a ubiquitous non-sulfated glycosaminoglycan (GAG) in the extracellular matrix^27^ extensively reported to be asociate with multiple solid malignancies^28,29^. HA is synthesized by 3 isoforms of transmembrane hyaluronan synthases (HAS), while HA degradation is carried out by isoforms of hyaluronidases (HYAL)^30^. In addition, the main receptor for HA is CD44, a marker of CSC as was described previously^31^. The interactions between HA and tumor cells drive the CSC phenotype and promote resistance to cisplatin in head and neck squamous cell carcinoma models^32^. Moreover, high expression of HA in the stroma of lung adenocarcinoma tumor samples has been associated with more frequent disease recurrences^33^, but the mechanism underlying this association remains unclear.

We have previously demonstrated that the coumarin derivative 4-methylumbelliferone (4Mu), also known as hymecromone, is a selective inhibitor of HA synthesis^34^ and reduces CSC properties in hepatocellular carcinoma^35^. In this work, we aimed to study whether targeting HA from CSC with 4Mu, could revert resistance induced by taxane-based chemotherapy.

## Results

### HA metabolism-associated molecules are differentially expressed in tumor samples from NSCLC patients and correlate with CSC transcription factors and markers.

**Figure 1:**
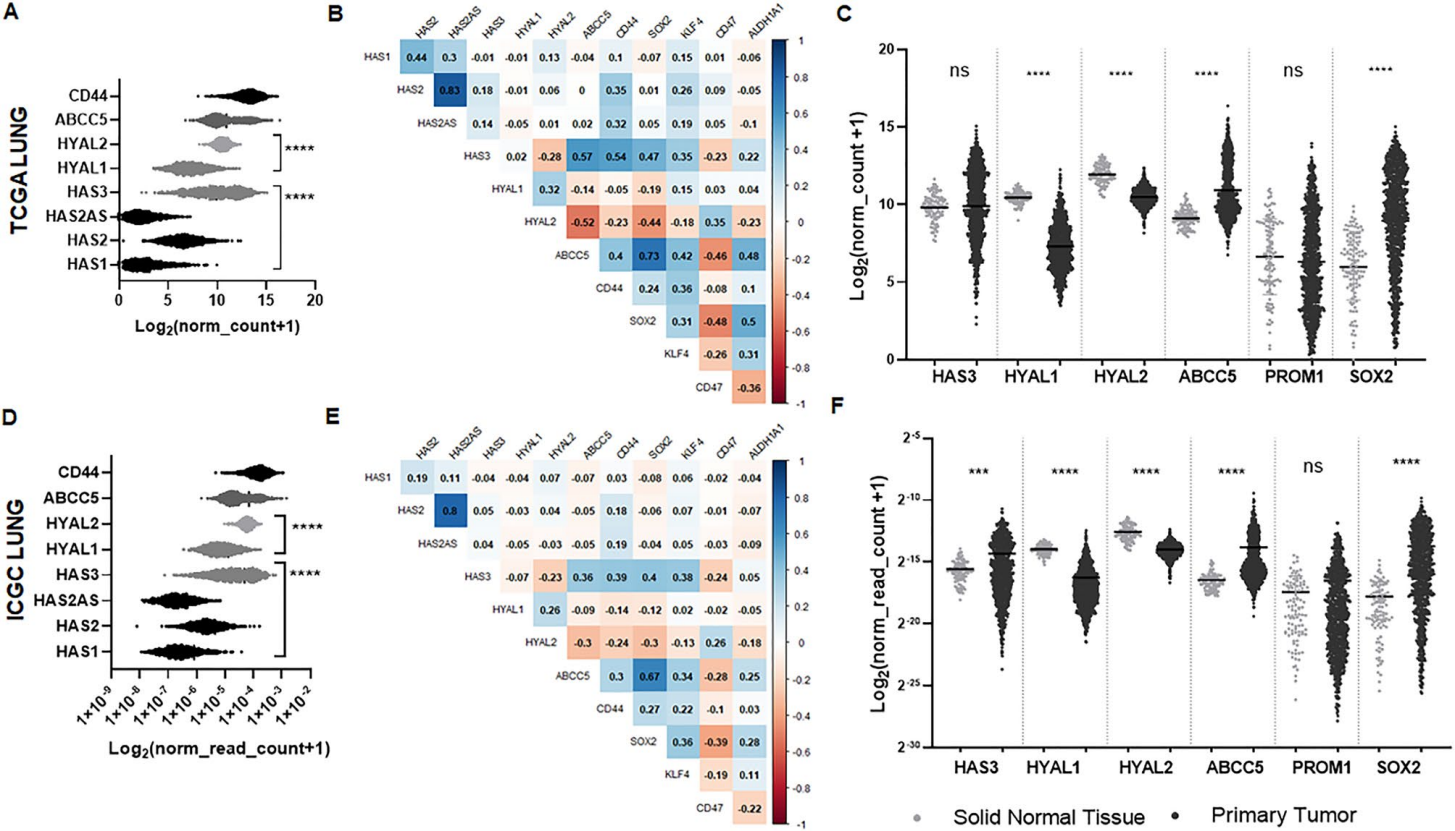
Expression of HA-related molecules and CSC markers in tumor tissue from NSCLC patients. (**A** and **D**) Expression of genes regulating hyaluronan synthases HAS1, HAS2, HAS2-AS and HAS3, hyaluronidases HYAL1 and HYAL2, ABCC5 and CD44 receptor obtained from TCGA and ICGC databases, respectively. Patient RNA-seq data is graphed as log2 (norm_count + 1) for TCGA and log2 (norm_read_count + 1) for ICGC, ****p < 0.0001 HAS3 versus HAS2, HAS2-A2 and HAS1; ****p < 0.0001 HYAL2 versus HYAL1, Kruskal–Wallis test. (**B** and **E**) Correlation heatmap of Pearson r values between HA- and CSC-related genes was created using RStudio software (R v 4.3.2 (https://www.r-project.org/, R package ‘corrplot’) and data obtained from TCGA and ICGC databases, respectively. (**C** and **F**) HAS3, HYAL1, HYAL2, Prom-1, ABCC5 and SOX2 expression in tumor tissue (TT) versus non-tumoral adjacent tissue (NTAT), obtained from TCGA and ICGC databases, respectively. ****p < 0.0001 HYAL1, HYAL2, ABCC5 and SOX TT vesus NTAT, Paired t test (TCGA); ****p < 0.0001 HAS3, HYAL1, HYAL2, ABCC5 and SOX TT versus NTAT, Paired t test (ICGC).

We explored a possible association between HA and CSC phenotype in NSCLC tumors. We analyzed mRNA expression from enzymes and molecules related to HA metabolism, such as synthesis, degradation, and export, employing the public databases TCGA LUNG and ICGC (lung cancer specimen). We first analyzed HA synthases HAS1, HAS2, and HAS3, hyaluronidases HYAL1 and HYAL2, ABCC5 transporter, and CD44 expression in tumor tissue (TT) in TCGA database (Fig. 1A). We also analyzed HAS2-AS1 gene expression, since it has emerged as an important regulator of HAS2 gene expression and HA production as well as survival and apoptosis^36^. We observed that HAS3 expression was higher than HAS2, HAS2-AS1, and HAS1 (*p* < 0.0001), while HYAL2 was more expressed than HYAL1 (*p* < 0.0001). ABCC5 transporter and CD44 also showed high expression levels. We then explored if there was an association between HA metabolism-associated molecules and markers or transcription factors characteristic of the CSC phenotype. We performed correlation analyses and, interestingly, we found a strong positive correlation between HAS3 and SOX2 (r = 0.47), KLF4 (r = 0.35), and ALDH1A1 (r = 0.22) (Fig. 1B). The ABCC5 transporter showed the same tendency, as it positively correlated SOX2 (r = 0.73), KLF4 (r = 0.42), and ALDH1A1 (r = 0.48) (*p* < 0.001) (Fig. 1B). Conversely, hyaluronidase HYAL2 inversely correlated with SOX2 (r = − 0.44) and ALDH1A1 (r = − 0.23) (Fig. 1B). Moreover, we found that HAS3 expression is strongly correlated with ABCC5 HA transporter (r = 0.57) and CD44 (r = 0.54) (Fig. 1B), which is the main HA receptor. Figure 1B also shows a more detailed book of maps of gene correlation between HA-metabolism molecules and CSC features. To contrast these results, we analyzed these correlations in the ICGC database and, notably, we observed similar results with the same tendency as the TCGA database (Fig. 1D and 1E).

Next, we analyzed the expression of all these HA metabolism-associated molecules and CSC-related factors or markers in both tumoral tissue (TT) and non-tumor adjacent tissue (NTAT) from the TCGA LUNG database. Interestingly, ABCC5 expression was higher in TT in comparison with NTAT (*p* < 0.0001), as well as SOX2 expression (*p* < 0.0001), and HAS3 expression (*p* < 0.001; ICGC database). In addition, we found significant lower expression of HYAL1 and HYAL2 in TT in comparison with NTAT (*p* < 0.001 and *p* < 0.001, respectively) (Fig. 1C). When we contrasted these results in the ICGC database, we observed similar results with the same tendency as in the TCGA database (Fig. 1F).

### Lung CD133^+^ CSC are tumor-initiating cells, which exhibit differential expression of HA and higher levels of CSC molecules involved in stemness phenotype.

**Figure 2:**
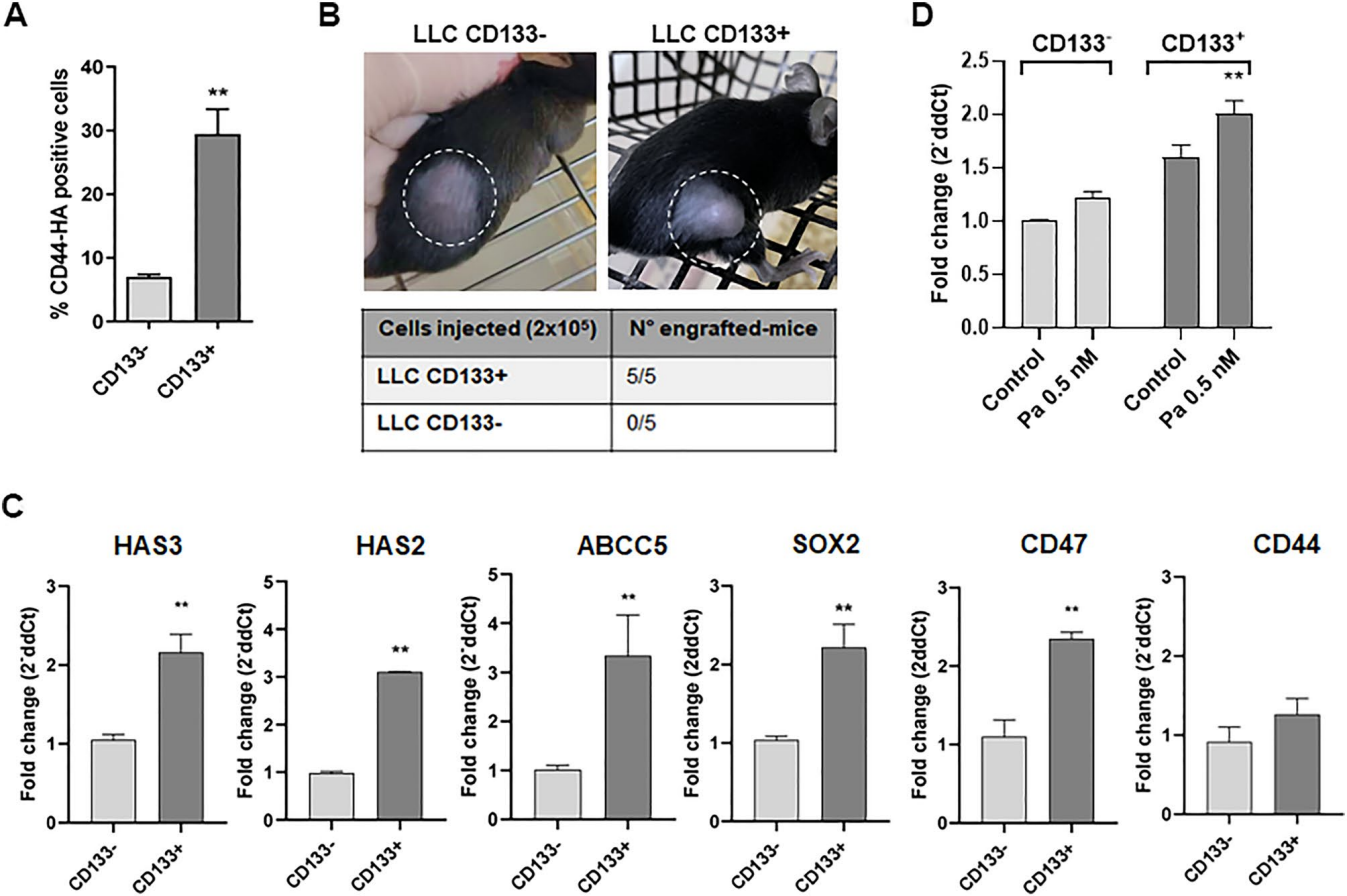
CSC marker expression and tumor-initiating ability of CD133 + LLC cells. (**A**) Levels of HA in CSC. Percentages of CD133-HA + CD44 + and CD133 + HA + CD44 + LLC cells. Magnetically isolated CD133 + and CD133- cells were stained with biotinylated HA-binding protein (+ HAbP) and avidin-FITC. **p < 0.01, Mann–Whitney test. (**B**) LLC CD133 + capability to induce tumors. Female C57BL/6 mice were injected with 2 × 10^5^ LLC CD133^−^ or CD133 + cells in the right flank. On day 15 post-inoculation only the mice injected with LLC CD133 + cells induced tumor appearance (n = 5/5). (**C**) Gene expression of HA metabolism-associated molecules and CSC factors or markers determined through RT-qPCR in magnetically isolated LLC CD133− and CD133 + cells. **p < 0.01, Mann–Whitney test. (**D**) ABCC5 gene expression in CD133 + and CD133− LLC cells following 24 h incubations with paclitaxel (Pa) 0.5 nM. **p < 0.01, Mann–Whitney test.

To validate in vitro and in vivo the findings observed in NSCLC patients, we first determined the presence of HA in the microenvironment of tumor sections from syngeneic tumor-bearing mice and the proportion of HA expressed in a murine cell line of lung cancer (LLC). We challenged mice with LLC cells and HA expression was assessed in samples from tumor-bearing mice. We observed positive staining of the glycosaminoglycan in vivo. We then confirmed through flow cytometry that HA observed in tumors is, at least in part, produced by LLC cells (9.2% ± 0.9%; Supplementary Fig. 1A ,B). We also found that whole LLC (wLLC) cells expressed the cancer stem marker CD133 (6.8% ± 0.4%; Supplementary Fig. 1C). It has been widely reported that CD133 is a reliable marker for lung CSCs by its tumorigenic capability to proliferate indefinitely, forming tumor spheres in a serum-free medium, generating an expansive progeny of non-tumorigenic cells and promptly producing tumor xenografts closely resembling the original tumor in immunocompromised mice^15,37,38^. Then, employing anti-CD133 microbeads and magnetic columns, we separated LLC cells into CD133^+^ and CD133^−^ fractions. Interestingly, when assessing the expression of HA in both fractions, we found that the CD133^+^ cells expressed significantly more HA (bound to CD44) than the CD133^−^ cells (34% ± 5.3% vs. 6.5% ± 1.3%; Fig. 2A). To confirm that the CD133^+^ cells corresponded to CSC, we evaluated their tumor-initiating capacity in vivo by inoculating them subcutaneously in C57BL/6 mice. We observed that while mice challenged with CD133^+^ developed tumors (n = 5/5), none of the mice inoculated with CD133^−^ cells showed tumor growth (Fig. 2B). We also analyzed the mRNA expression of HA metabolism-associated molecules and CSC factors and markers in CD133^+^ cells compared with CD133^−^ cells. Notably, we found that CD133^+^ cells showed higher levels of HAS2, HAS3, and ABCC5 gene expression together with a higher expression of CD47 and SOX2 in comparison with CD133^−^ cells (p < 0.01). In addition, the expression of CD44 showed no differences between CD133^+^ and CD133^−^ cells (Fig. 2C). Regarding the reported evidence that CSC become resistant to conventional chemotherapeutic drugs such as taxanes, we cultured CD133^+^ and CD133^−^ cells with paclitaxel (Pa) and observed an increase in ABCC5 gene expression in the CD133^+^ population compared to CD133^−^ cells (p < 0.05 respectively; Fig. 2D). Moreover, we found CD133^+^ cells to be more resistant to Pa chemotherapy than the wLLC and CD133^−^ populations (Fig. 4A).

### HA modulation in lung *cancer* cells by coumarin derivative 4-methylumbelliferone confers sensitivity to paclitaxel.

**Figure 3:**
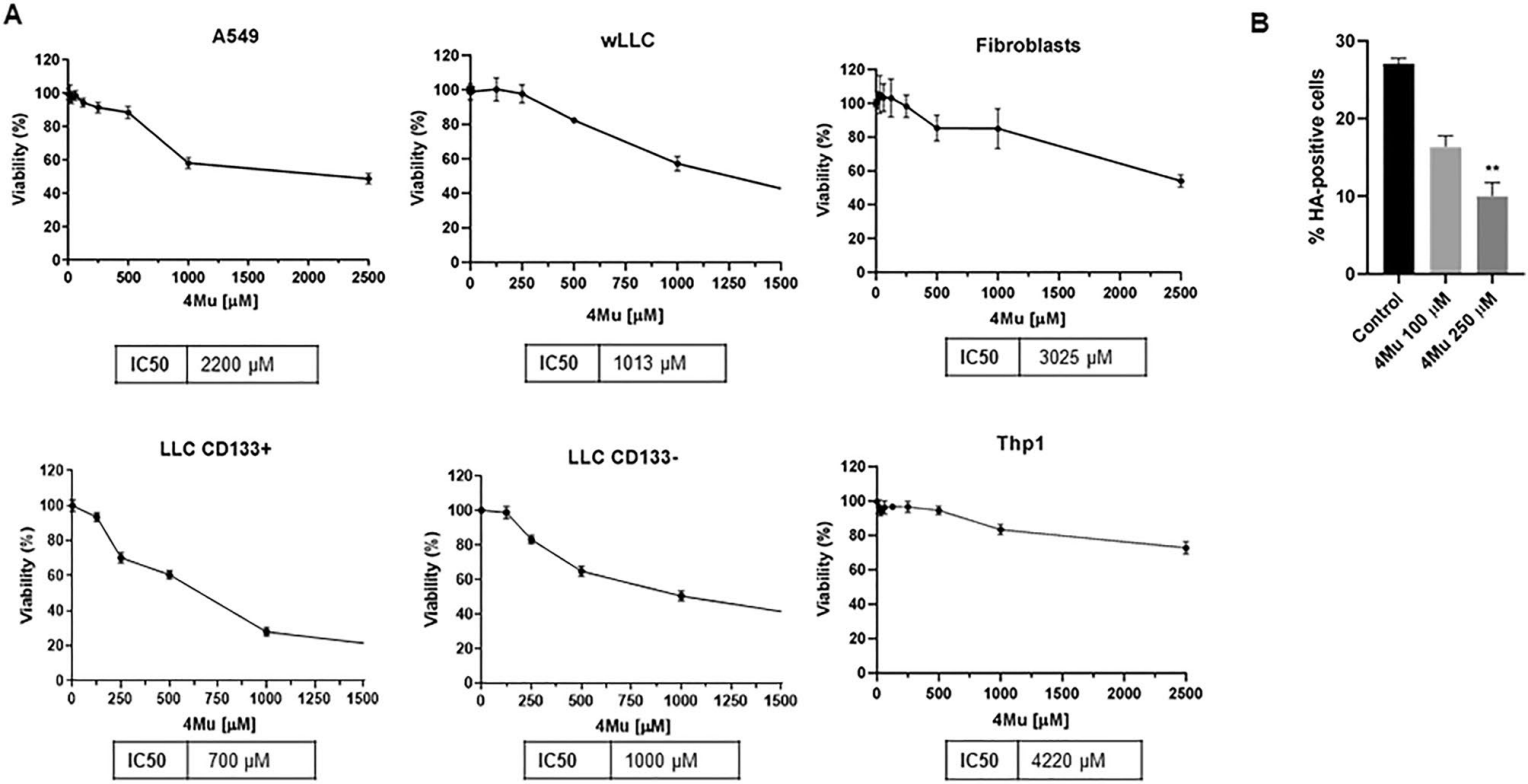
Effects of 4Mu on viability and HA synthesis in human and murine cell lines. (**A**) Viability assays after exposure to different doses of 4Mu for 72 h in human A549 lung cancer cells, murine LLC lung cancer cells and non-tumor cells. IC50 values were obtained with Prism Software. (**B**) Inhibition of HA expression by 4Mu on LLC cells. The percentage of HA + cells was determined with BD Accuri software. **p < 0.01 versus Control, Kruskal–Wallis test.

Elevated HA secretion has been reported in different solid tumors including renal, colorectal, ovarian, breast, and lung cancer^28,39^. We and others have previously demonstrated that nutraceutical coumarin derivative 4-methylumbelliferone (4Mu) is capable of selectively modulating the synthesis of HA through the reduction of HAS mRNA levels and by depletion of the HA precursor UDP-glucuronic acid^34^, leading to different effects both in cancer cells and in the TME^40^. Intending to evaluate this capability on lung cancer cells, we first assessed the viability of A549, wLLC, CD133^+^ LLC, CD133^−^ LLC, fibroblasts, and Thp1 human monocytes after exposure to different doses of 4Mu (Fig. 3A). We observed that lung cancer cells were affected from 1000 to 2200 μM, while this dose does not affect non-tumor cells. In addition, we observed that CD133^+^ cells were more sensitive to 4Mu compared to CD133^−^ cells. Then, we analyzed HA inhibition by sublethal doses of 4Mu using flow cytometry. We observed a significant reduction of HA expression starting at 4Mu 250 μM (62% inhibition vs. control; *p* < 0.01; Fig. 3B).

**Figure 4:**
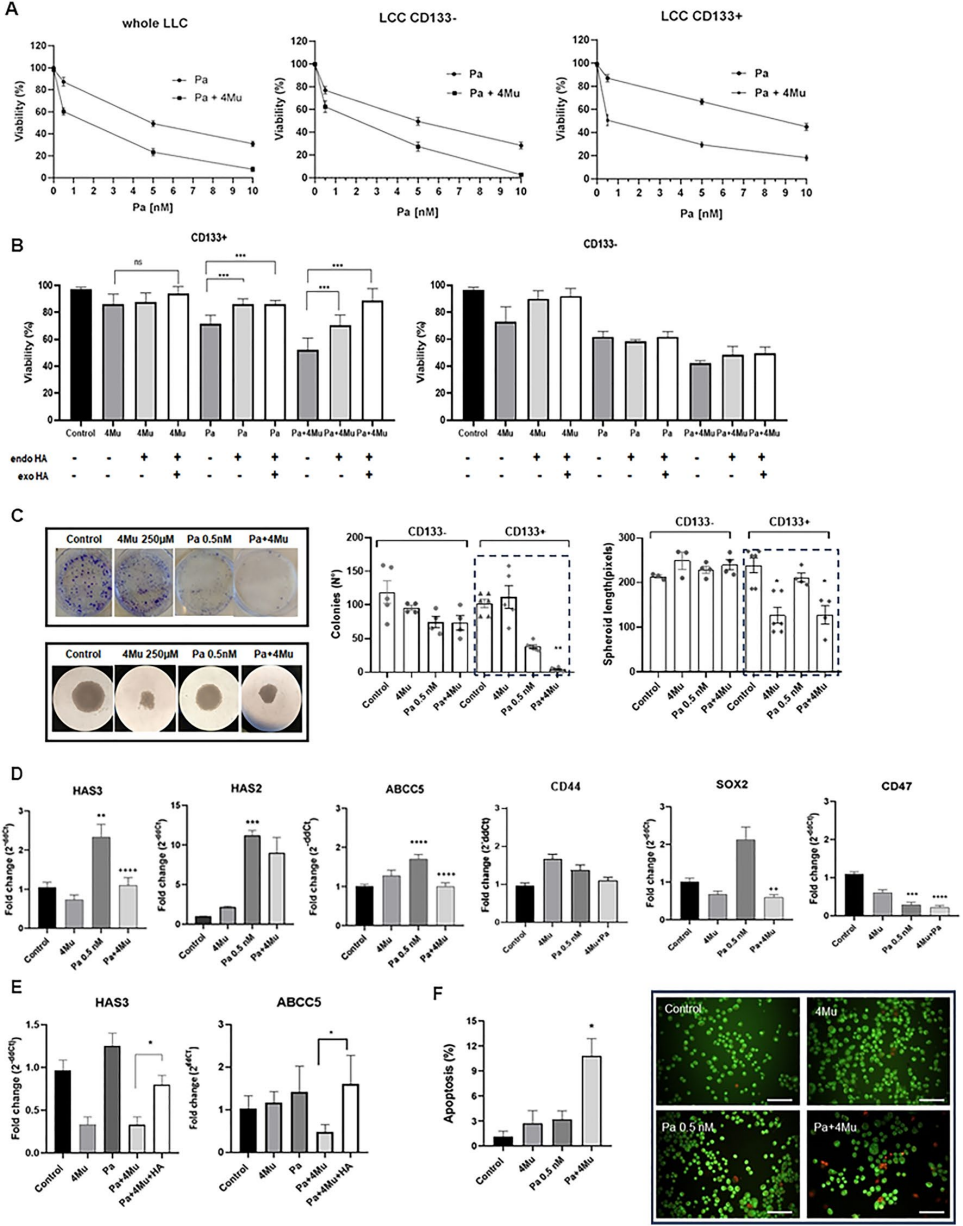
4Mu impairs stemness marker expression, colony formation and viability of murine LLC cells when combined with paclitaxel (**A**) Viability assays in whole, CD133^−^ and CD133^+^ LLC cells, performed after 72 h incubations with 4Mu 250 μM and various concentrations of paclitaxel. (**B**) Viability assays in CD133^−^ and CD133^+^ LLC cells, performed after 48 h post-treatment withdrawal. The incubations with 4Mu 250 μM and 2 nM paclitaxel were assessed during 72 h. Then, treatment was withdrawn and cells were incubated with culture medium (endogenous HA; endoHA) or culture medium + exogenous 200 µg/ml HA (exoHA); ***p < 0.001 CD133 + Pa versus Pa + endoHA and versus Pa + endoHA + exoHA; ***p < 0.001 CD133 + Pa + 4Mu versus Pa + 4Mu + endoHA and versus Pa + 4Mu + endoHA + exoHA, Tukey multiple comparison test. (**C**) Clonogenic assays were performed with CD133^−^ and CD133^+^ LLC cells, treated or not with 4Mu 250 μM and/or paclitaxel (Pa) 0.5 nM. *p < 0.05, **p < 0.01 versus Control, Kruskal–Wallis test. Three-dimensional spheroid assays for CD133^+^ and CD133^−^ LLC cells treated or not with 4Mu 250 μM and/or Pa 0.5 nM. The spheroid diameter was measured using ImageJ software (NIH). **p < 0.01, p*** < 0.001, p**** < 0.0001 versus Control. ++*p* < 0.01, ++++*p* < 0.0001 versus Pa, Kruskal–Wallis test. (**D**) Gene expression levels of HA metabolism-associated molecules and CSC-related factors in LLC cells. Gene expression was determined through RT-qPCR in cells treated or not with 4Mu 250 μM and/or Pa for 24 h. *p < 0.05, ***p < 0.001 versus Control, Kruskal–Wallis test. (**E**) Gene expression levels of HAS3, ABCC5 and CD47 in CD133 + cells cultured in the presence of exogenous HA. Gene expression was determined through RT-qPCR in cells treated or not with 4Mu 250 μM and/or Pa for 24 h. After treatment, culture medium was replaced and supplemented with 200 µg/ml HA for 48 h HAS3, ABCC5 *p < 0.05, 4Mu + Pa versus 4Mu + Pa + HA. Kruskal–Wallis test. (**F**) *Right:* Apoptosis analysis of LLC cells at 24 h of incubation with 4Mu 250 μM and/or Pa 0.5 nM, performed by acridine orange and ethidium bromide staining. Scale bar 100 µm. *Left:* Quantification of apoptosis assay was done by calculating the percentage of apoptotic cells in at least 100 cells per treatment. *p < 0.05 versus Control, Kruskal–Wallis test.

Then, we aimed to test if HA inhibition by 4Mu could increase the sensitivity of lung cancer cells to chemotherapy. We assessed viability in CD133^+^, CD133^−^ and wLLC after taxane (Pa) exposure at different doses, combined with 4Mu 250 μM. We observed that 4Mu increased LLC sensitivity to Pa (ranging from 0.15 nM to 10 nM) especially in CD133^+^ cells (Fig. 4A). In addition, when we withdrew the treatments for 24 h and performed the cell survival assay, we detected that the presence of endogenous HA overcome the viability decrease induced by Pa and 4Mu + Pa (p < 0.001). We obtained a similar behaviour when we added exogenous HA (p < 0.001; Fig. 4B). To analyse the functional impact of chemotherapy combined with HA inhibition, we performed colony formation and 3D spheroid assays. We found that the chemotherapeutic drug (0.5 nM) combined with 4Mu significantly reduced colony formation in CD133^+^ cells (*p* < 0.01; Fig. 4C) and inhibited tumor-formation capacity in CD133^+^ cells (*p* < 0.05; Fig. 4C). Considering that platinum-based antineoplastics are currently used with immunotherapy or taxanes for patients with advanced NSCLC, we also tested the effect of a combination of 4Mu and cisplatin. We observed similar results about the capability to inhibit colony and 3D spheroids formation in the CD133^+^ population employing a dose of 0.125 nM of cisplatin (p < 0.05; Supplementary Fig. 2A,B). Importantly, we also observed the capability of anchorage-independent cell growth of CD133 + cells in a soft-agar colony formation assay (Supplementary Fig. 2C).

Next, we explored the effects of Pa + 4Mu on the expression of genes related to HA metabolism and CSC phenotype. We found that Pa significantly increased mRNA transcription of HAS2, HAS3, ABCC5, and SOX2 after 24 h of exposure (p < 0.05; Fig. 4D). Importantly, when LLC cells were treated with Pa + 4Mu combination, mRNA levels significantly decreased (p < 0.01), while CD44 receptor expression did not show significant changes with any treatment. We then examined the effect of the addition of exogenous HA and detected that the reduced levels of HAS3 and ABCC5 induced by combined treatment in CD133 + were reverted (Fig. 4E). We observed a similar result in gene expression after treatment with 4Mu, alone and combined with Pa, in human lung cancer cell line A549 (Supplementary Fig. 3A). Thus, 4Mu downregulates the transcription of molecules implicated in the stem cell phenotype, contributing to overcoming resistance to chemotherapy. Moreover, the percentage of apoptotic cells measured by acridine orange staining after 72 h of Pa + 4Mu combination therapy was significantly higher than after exposure to single agents (p < 0.05; Fig. 4F).

### 4Mu improves paclitaxel chemotherapy outcomes in a murine model of NSCLC

**Figure 5:**
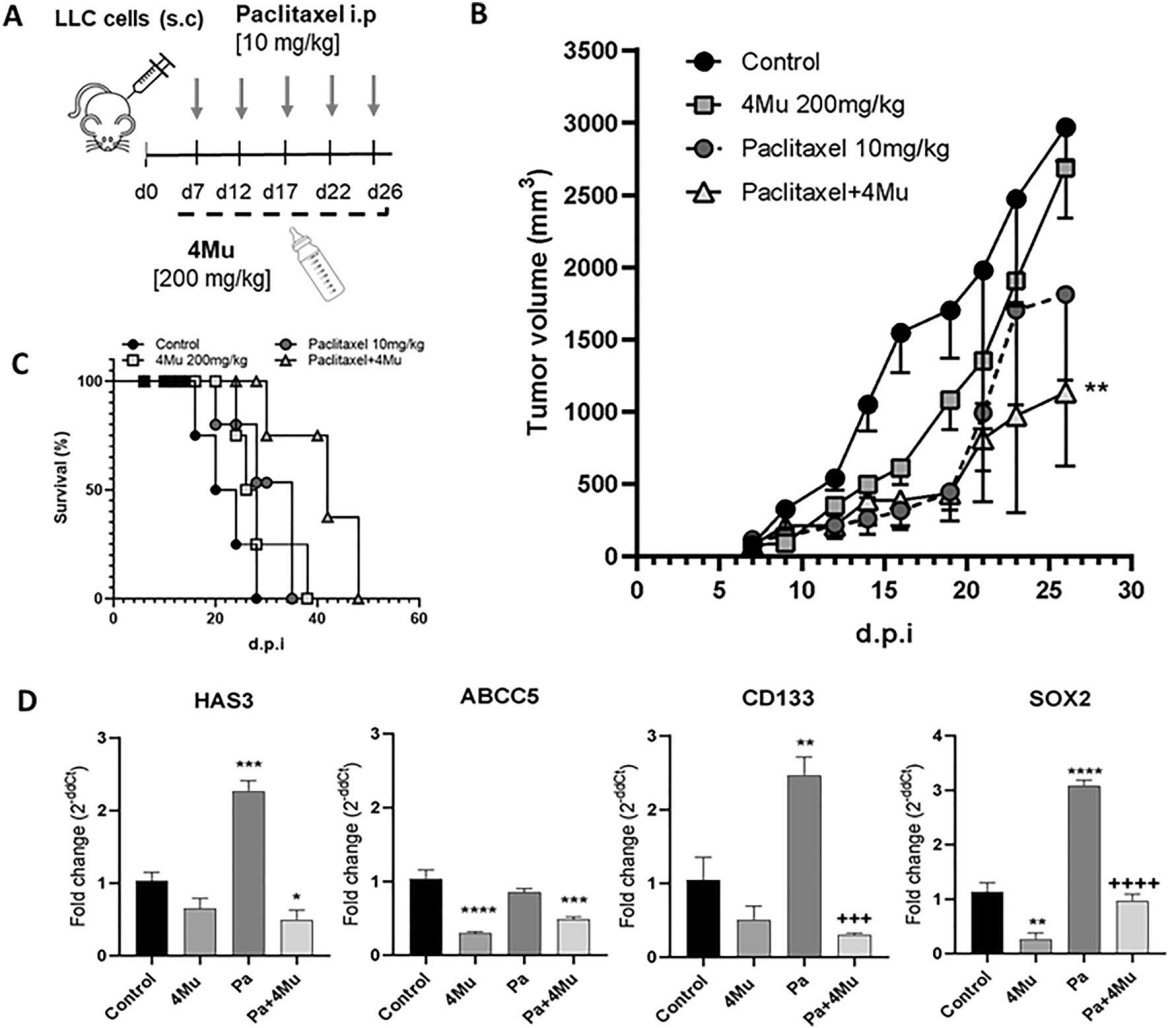
In vivo effects of 4Mu combined with paclitaxel on tumor growth, gene expression, and survival in a murine model of NSCLC. (**A**) Experimental design of the murine model of NSCLC. 5-week-old C57BL/6 females were used (n = 5). On day 7 post-inoculation, when the tumors reached 80 mm^3^, 4Mu was administered in the drinking water ad libitum and paclitaxel intraperitoneally (i.p.) every 5 days. (**B**) Tumor growth curve. The combination of Pa + 4Mu significantly reduces tumor volume. **p < 0.01 versus Control, ANOVA Test. (**C**) Kaplan–Meier survival curve of LLC tumor-bearing mice. (**D**) Expression of HAS3, ABCC5, CD133, and SOX2 mRNA in murine tumor tissue. *p < 0.05, **p < 0.01, ***p < 0.001 versus Control. + + +*p* < 0.001, ++++*p* < 0.0001 versus Pa, Kruskal–Wallis test. d.p.i., Days post-inoculation.

To assess the in vivo effect of the combination of HA inhibition with Pa, we treated LLC tumor-bearing C57BL/6 mice with 4Mu and/or Pa in a schedule similar to clinical practice schemes^41^ (Fig. 5A). Mice treated with Pa + 4Mu showed significant inhibition of tumor growth compared to control mice (p < 0.01; Fig. 5B). In addition, mice treated with Pa alone showed a partial response at the beginning of treatment; however, after fifteen days of therapy, tumors progressed similarly to control mice. Pa + 4Mu combination therapy also induced a significant improvement in survival of treated mice (*p* < 0.01, Fig. 5C). Notably, the analysis of HAS3, ABCC5, SOX2 and CD133 tumor mRNA transcripts showed that Pa + 4Mu combination therapy significantly downregulated gene expression, as was observed in the previous in vitro assays (*p* < 0.01; Fig. 5D). Histopathology analysis revealed no architectural alterations in lung, liver, heart, and kidney tissue sections attributable to acute paclitaxel toxicity at 10 mg/kg doses (Supplementary Fig. 3B).

**Figure 6:**
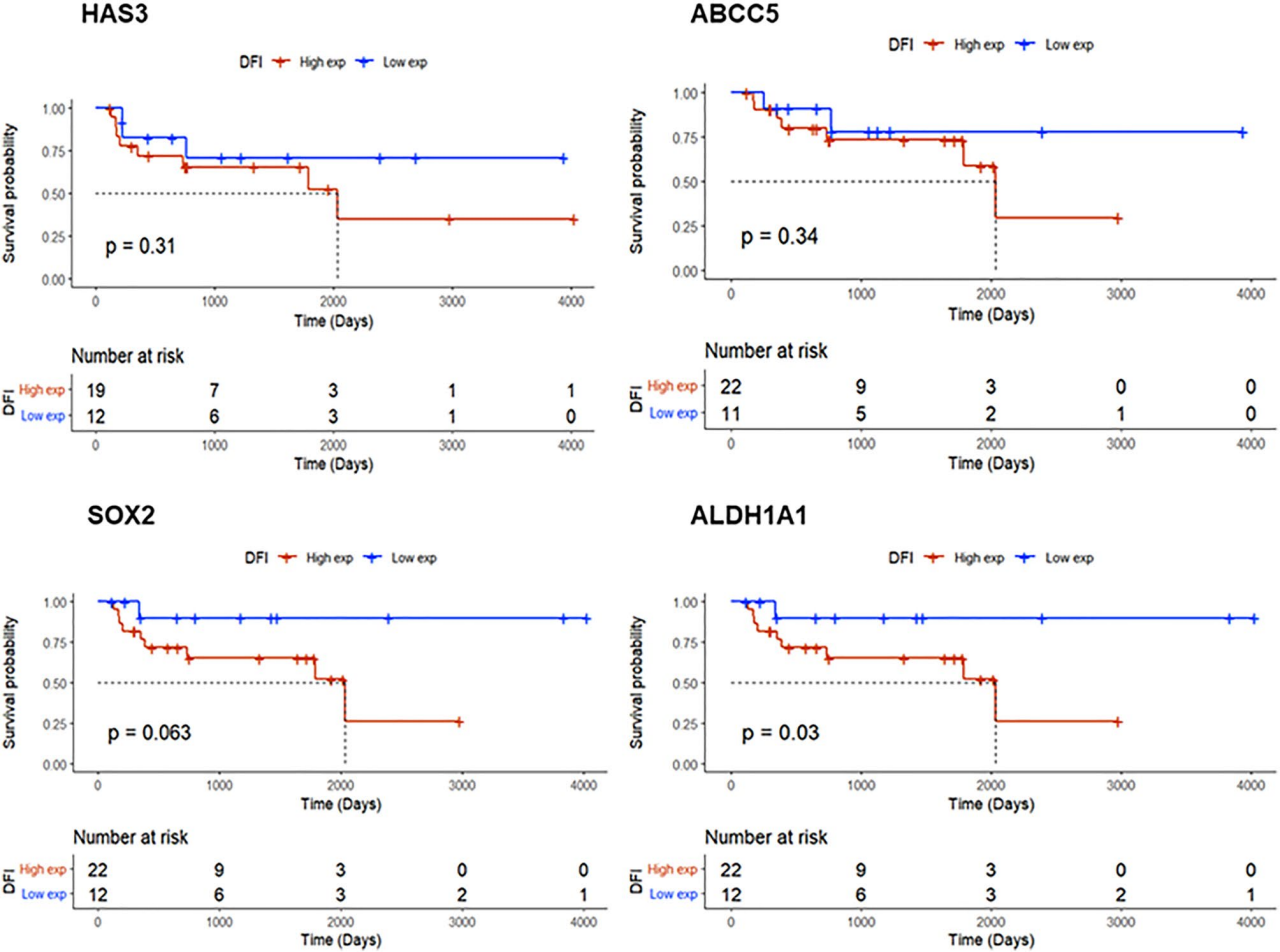
Kaplan-Meyer analyses for HAS3, ABCC5, SOX2, and ALDH1A1 expression in advanced-stage lung cancer patients (stages IIIA, IIIB, and IV). Expression levels were divided into quartiles and cut-off values are indicated in each graph. Data was obtained from TCGA Lung Cancer. Red, high expression; Blue, low expression; DFI, disease-free interval.

Subsequent to our in vivo results concerning the ability of 4Mu + Pa to induce a decrease of HA metabolism and CSC phenotype-related transcripts in tumor samples from treated mice, we ultimately explored the impact of a dysregulated expression of these molecules in tumor samples from patients with NSCLC stages IIIA, IIIB and IV. The analysis of TCGA showed that patients with a higher expression of HAS3, ABCC5, SOX2, and ALDH1 could experience a shorter disease-free interval (DFI), a prognostic factor defined as the interval from the completion of chemotherapy to the diagnosis of disease recurrence (Fig. 6).

## Discussion

Despite the major changes in lung cancer approaches over the last decade and focused efforts on prevention and early diagnosis, this type of cancer is still the leading cause of cancer-related deaths^1^. Drug resistance is a major cause of conventional therapy failure, in which CSC play a key role due to their self-renewal, tumor-initiating abilities and metastatic potential^42^. Furthermore, the presence of CSC has been described in different tumors, such as lung, thyroid, colorectal, breast, prostate, and other solid tumors^43^. For that reason, the sensitisation of CSC to conventional treatment remains a promising field of study.

It has been reported that interactions between tumor cells and HA can induce changes toward a CSC phenotype^32^ and promote resistance to cisplatin in head and neck squamous cell carcinoma models^44^. HA is a ubiquitous glycosaminoglycan (GAG) in the extracellular matrix^27^ and is overexpressed in different types of tumors. In lung adenocarcinoma, HA expression in stroma was associated with more frequent disease recurrences^45^.

We first made a screening in public databases TCGA and ICGC, analyzing gene expression of molecules implicated in HA metabolism in tumors from lung cancer patients. We found that HA synthases HAS2 and HAS3 were differentially expressed and, interestingly, that their expression was associated with CSC phenotype markers. In addition, ABCC5 emerged from the databases’ exploration, and we found that it was highly expressed in tumors from patients with lung cancer and strongly correlated with the CSC phenotype. A recent analysis of the molecular dynamics of a viral HAS homolog proposed that vertebrate HAS2 is structurally and functionally capable of extruding HA^46^, and a previous report also demonstrates the role of ABCC5 in the extracellular export of HA^47^. This, coupled with our results, suggests that cells with CSC phenotype in lung cancer could produce and transport HA to the extracellular matrix.

We then assessed HA expression by NSCLC cells, both in vitro and in vivo, in an experimental model in mice induced by subcutaneous injection of murine LLC cells. We also determined that about 7% of whole LLC cells were positive for CD133 (CD133^+^) and attributed this subpopulation to CSC since CD133 is a distinctive marker of them. Then, we separated CD133^+^ and CD133^−^ fractions from the whole LLC, and notably found that lung CD133^+^ cells differentially expressed HA, supporting the association between HA-associated molecules and some CSC markers that we obtained from the analysis of lung cancer patients’ data. We confirmed that the CD133^+^ fraction had tumor-initiating capacity, a characteristic property of CSC, by inducing tumors in vivo in syngeneic C57BL/6 mice. In addition, we also observed in vitro that CD133^+^ cells differentially expressed other CSC markers such as CD47 and the transcription factor SOX2, in comparison to CD133^−^ cells. Moreover, we determined that HAS2, HAS3 and the transporter ABCC5 were overexpressed in CD133^+^ cells. The relationship between HA, CSC profile, tumor persistence and chemoresistance has been previously described in association with its principal receptor CD44 in many types of solid tumors, such as human head and neck squamous cell carcinoma (HNSCC)^48^. Bourguignon et al., have widely demonstrated that a high level of CD44 with ALDH1 detected in the subpopulation of human HNSCC-CSC display the hallmark of stem cell properties, including NANOG, OCT4, and SOX2 expression, self-renewal capability and high tumorigenic potential in immunosuppressed mice^44^. In addition, previous studies showed that HA/CD44 promotes cancer cell proliferation and tumor progression^49^. Although the HA/CD44 axis and their role in lung tumorigenesis have been previously described, we reported for the first time a correlation between HA and CSC phenotype, together with a role in cancer progression and clinical outcomes since patients with a higher expression of HAS3, HAS2, ABCC5, SOX2, and ALDH1 showed a decrease in their disease-free intervals.

We also hypothesized that HA produced by CSC is implicated in chemoresistance in lung cancer and we were interested in its modulation to make CSC more sensitive to chemotherapy. 4-methylumbelliferone (4Mu), also known as hymecromone, is a coumarin derivative that has been safely used in humans as a choleretic orally, and studied in clinical trials as well^50^. It has been reported that the main proposed mechanism for the inhibitory effect of 4Mu on HA synthesis is the depletion of the HA precursor UDP-glucuronic acid. 4Mu can also modulate HA metabolism through the inhibition of HAS2 and HAS3 transcripts^34^. Other mechanisms reported in different types of tumors include the promotion of caspase-dependent apoptosis, cellular arrest, reduction of proliferation and pro-angiogenic factors, as well as modulation of inflammatory and oxidative factors^51^. Moreover, Doshi et al. have shown that UDP-glucuronic acid could be critical to cancer cell metabolism^52^ and the reduction of this HA-precursor production affects detoxification. This reflects a key role of UDP-glucuronic acid^53^ and also a possible role in chemotherapeutics drugs metabolism that could promote an increase in chemotherapy sensibility. Some studies have reported 4Mu effects on chemotherapy, showing a stronger anti-tumoral effect in comparison with chemotherapy alone in ovarian cancer and glioblastoma^54,55^. To assess if HA inhibition by 4Mu was able to reduce NSCLC susceptibility to chemotherapy, we performed viability assays combining 4Mu with the taxane paclitaxel. Interestingly, LLC CD133^+^ cells showed higher sensitivity when treated with the combination of 4Mu and paclitaxel (Pa + 4Mu) compared to Pa alone, in contrast to CD133^−^ cells. In functional assays, the combination Pa + 4Mu drastically reduced the number of colonies in a clonogenic assay and spheroid length, which represent the tumor-initiating capacities of CSC, only in CD133^+^ cells. Similar results were obtained when we treated lung cancer cells with cisplatin + 4Mu.

We also observed that paclitaxel induced the overexpression of HAS2, HAS3, ABCC5 and SOX2 in LLC cells, and when combining Pa with 4Mu, the expression of HAS3, ABCC5, CD47 and SOX2 reverted to levels similar to those seen in controls. According to these results, we have previously reported that 4Mu strongly reduces the expression of stemness markers such as CD47 and SOX2^35^. In addition, the expression of CD47, which is involved in CSC’ ability to evade the immune system^56^, was also significantly reduced by Pa + 4Mu. CD47 expression on CSC from human solid tumors is considered a mechanism of phagocytosis evasion, as a means to overcome immune recognition and allow tumor progression^57^. The capability of our combination to reduce CD47 expression could become substantial in enabling successful treatment since advanced NSCLC patients without driver mutations are currently managed with chemotherapy plus immunotherapy. Similar results were observed in human A549 cells, confirming that the observed effects are independent of the cell line.

One of our hypotheses that could explain the induction of HA synthesis by HAS2 and HAS3, as well as by ABCC5, is that it is a result of chemoresistance. It is known that the ABC family of proteins contributes to chemotherapy resistance since they are drug-transporter pumps, and they are overexpressed in CSC^58^. In addition, it was shown in nasopharyngeal cancer cells that paclitaxel resistance is regulated by the FOXM1-ABCC5 axis^59^. Although recently and using computational modelling it has been postulated that HAS opens a transmembrane channel to coordinate hyaluronan synthesis and export HA outside the cell^46^, it was also described that ABCC5 participates in the extrusion of HA^47^. This multidrug resistance receptor was also reported in lung cancer cells^60^. Here we showed that ABCC5 and HAS3 are overexpressed in lung CD133^+^ CSC and more importantly, the Pa + 4Mu strategy was able to modulate both HAS3 and ABCC5 transcript levels back to basal expression. Mammalian cells synthesize HA in the cytoplasm and actively release it into the microenvironment through energy-dependent transport proteins. Inhibition of various transport proteins led to a reduction in HA export, and it has been reported that the inhibition of the ABCC5 transporter proved to be the most effective in decreasing HA levels. This suggests that hyaluronan export is a key physiological function of the ABCC5 transport protein. In line with this result, another natural compound, the curcumin analogue *hyalin* was identified as an inhibitor of HA export by virtual docking to the ABCC5 transporter^61^.

Importantly, we demonstrated the capability of 4Mu to improve paclitaxel chemotherapy outcomes and significantly reduce tumor growth in lung cancer-bearing mice. This result was also reflected in a higher survival rate. Moreover, the reduction of HAS3, ABCC5, and SOX2 gene expression induced by Pa + 4Mu, previously observed in vitro, was confirmed in tumor tissue from treated mice. Additionally, CD133 gene expression was also reduced, indicating that the CSC phenotype was impaired. This effect is in line with the fact that HA inhibition by 4Mu made both whole LLC and CD133^+^ cells more sensitive to chemotherapy.

## Materials and Methods

### Databases analysis

Gene expression (RNA sequencing [RNA-Seq]) for the indicated genes was obtained from The Cancer Genome Atlas (TCGA) for patients with lung cancer (TCGA Lung Cancer, N = 1325) using UCSC Xena software and International Cancer Genome Consortium (ICGC, N = 2207). Correlation analysis between genes was performed using RStudio software (R v 4.3.2, https://www.r-project.org/, R package 'corrplot'). R values > 0.2 were considered as positive. For tumor (T) versus non-tumoral adjacent tissue (NTAT) comparison, paired samples from both types of tissues were used to calculate the individual fold changes. Kaplan-Meyer analyses for specific gene expression were performed with data from TCGA, selecting patients in advanced stages of lung cancer. Data supporting reported results regarding patients can be found at https://xena.ucsc.edu/.

### Cell lines

Lewis Lung Carcinoma (LLC) cells (syngeneic with C57BL/6 mice) is a cell line from American Type Culture Collection (ATCC; CRL-1642) that were kindly provided by Dr. Ada Blinder (IByME-CONICET, CABA, Argentina). They have been tested for mycoplasma by the Polymerase Chain Reaction (PCR)-based detection procedure involving three steps: cell culture supernatant collection, DNA isolation, and PCR. LLC cells were grown in DMEM (Serendipia Lab, Vedia, Buenos Aires, Argentina) with 10% foetal bovine serum (FBS). A549 cells from ATCC (CCL-185) were kindly provided by Dr. Elizabeth Martinez (UT Southwestern, Dallas, Texas, USA). A549 cells also were tested for mycoplasma as we described above and grown in RPMI 1640 (GIBCO, Invitrogen Argentina, Buenos Aires, Argentina) with 10% FBS (Natocor, Córdoba, Argentina).

For in vitro studies, murine or human lung cancer cells (5 × 10^3^ per well) were seeded and treated with 4Mu at 125 µM, 250 µM, 500 µM or control vehicle solution (Hank’s balanced salt solution; HBSS) for an additional 72 h.

### Cell isolation by MACS

LLC cells were labeled with primary CD133 antibody (rat IgG1130-092-564, Miltenyi Biotec, Bergisch Gladbach, Germany), and the CD133^+^ cells were subsequently isolated using magnetic-activated cell sorting (MACS) columns. CD133^+^ cells were enriched using LS columns according to the manufacturer’s instructions. The purity of sorted cells was evaluated by flow cytometry. Trypan blue staining was used to assess cell viability.

### Flow cytometry

Briefly, 1 × 10⁶ isolated CD133^+^ or CD133^−^ cells were incubated with anti-mouse CD44-AlloPhycoCyanine (APC) (561,862, BD Biosciences) and a biotinylated HA-binding protein (HAbP) (Calbiochem) on ice for 45 min. Cells were washed thoroughly with PBS–1% bovine serum albumin (BSA), incubated with streptavidin-fluorescein isothiocyanate (FITC) (554,060; BD Biosciences) for 30 min, washed and subjected to flow cytometry (BD Accuri C6). Data analysis was performed with BD Accuri C6 software.

### Animals

6- to 8-week-old female C57BL/6 mice were maintained at our Animal Resources Facilities following the experimental ethical committee and the NIH guidelines on the ethical use of animals. The experimental protocol (19–13) was approved by the Animal Care Committee of the School of Biomedical Sciences, Universidad Austral, based on the essential points of the ARRIVE guidelines. For euthanasia, animals were deeply anaesthetized in a CO_2_ chamber and sacrificed by cervical dislocation.

### In vivo experimental model

C57BL/6 mice were subcutaneously inoculated with 2 × 10⁶ LLC cells into the left flank (day 0). When tumors were palpable and reached a volume of approximately 90 mm^3^, measured with a caliber, mice were homogeneously distributed in groups (n = 5–8 per group) and received: (1) saline (control); (2) 4Mu, 200 mg/kg drinking water; (3) Paclitaxel, 10 mg/kg intraperitoneally (i.p.), every 5 days; (4) Paclitaxel + 4Mu. Tumor volume was measured with a caliber 3 times per week and weight was registered once per week. Mice were sacrificed and samples from lung, liver, heart, kidney, and tumor were used for histopathology and RNA extraction. The experiment was carried out 3 times. For the tumor-inducing capacity of CD133^+^ LLC cells experiments, 2 × 10^5^ CD133 + or CD133^−^ cells were subcutaneously inoculated in C57BL/6 mice (n = 5/group), and tumor size was measured with a caliber as described above.

The animal study protocol was approved by the Institutional Ethics Committee of Instituto de Investigaciones en Medicina Traslacional (protocol code 19–13 11/6/2019).

### Histopathological analysis

Paraffin-embedded lung, liver, heart, and kidney tissue samples were stained with haematoxylin–eosin. Briefly, HA staining was performed in paraffin-embedded lung sections that were incubated with 3% H_2_O_2_–methanol for 30 min at room temperature (RT) to block endogenous peroxidase, followed by avidin, biotin, and protein-blocking solution Then, 5 μg/mL of bHA-BP diluted in 1% BSA–PBS was applied for 1 h. Negative controls were stained with bHA-BP and pre-treated with 100 U/mL of *Streptomyces* hyaluronidase at 37 °C for 30 min. Peroxidase complex (Sigma) 1:10 in PBS was used as a revealing system. The signal was detected by 0.1% diaminobenzidine 4% glucose, 0.08% ClNH_4_, and 5% nickel ammonium sulfate in 0.2 M AcNa 0.05% H_2_O_2_.

### RNA isolation and quantitative PCR analysis

Total RNA samples were isolated using TRI Reagent (Sigma-Aldrich, St. Louis, MO, USA), and total RNA (1 µg) was reverse transcribed (qRT-PCR) with 200 U SuperScript II Reverse Transcriptase (Invitrogen, Carlsbad, CA, USA) using 500 ng oligo(dT) primers. cDNAs were subjected to real-time qPCR (Stratagene Mx3005p, Stratagene, La Jolla, CA, USA). For qRT-PCR, the mRNA levels of HAS2, HAS3, ABCC5, CD133, CD44, CD47, and SOX2 were quantified by SYBR Green (Invitrogen, Carlsbad, CA, USA), using the following primers (Table 1):

**Table 1:** Specific primers used for RT-qPCR.

	Primer forward (5′-3′)	Primer reverse (5′-3′)
m-hGAPDH	CAGCGACACCCACTCCTCCACCTT	CATGAGGTCCACCACCCTGTTGCT
m-hHAS2	GGCCGGTCGTCTCAAATTCATC	CACAATGCATCTTGTTCAGCTC
m-hHAS3	CAAGCGTGAGGTCATGTACAC	TCCAGCACAGTGTCAGAGTC
mABCC5	CCGTTCCAGGCTGAGATGTT	GCGTGATATTGTCCAACCGC
mCD44	CACCTTGGCCACCACTCCTA	GGAGTCTTCACTTGGGGTAGGG
hCD44	GCGCAGATCGATTTGAATATAA	GTGCCCTTCTATGAACCCAT
mCD133	TCGGCATAGGGA AAGCCACG	GGGCACAGTCTCAACATCGTC
hCD133	AAACAGTTTGCCCCCAGGAA	ACAATCCATTCCCTGTGCGT
mSOX2	GATCAGCATGTACCTCCCCG	CGCCCTCAGGTTTTCTCTGT
hSOX2	CATGAAGGAGCACCCGGATTA	TTCATGTGCGCGTAACTGTC
mCD47	ATGCTTCTGGACTTGGCCTC	CCGACCAAAGCAAGGACGTA

*m* mouse, *h* human.

Amplifications were carried out using a cycle of 95 °C for 10 min and 45 cycles under the following parameters: 95 °C for 30 s, 56 °C for 30 s, and 72 °C for 1 min. At the end of the PCR reaction, values were normalised to levels of glyceraldehyde 3-phosphate dehydrogenase (GAPDH; used as housekeeping) transcript. Data were processed using the ΔΔCt method. The relative amount of the PCR product amplified from untreated cells (Control) was set as 1. A non-template control (NTC) was run in every assay, and all determinations were performed in three separate experiments. The relative expression was calculated according to the following equation: relative expression (RE) = 2^−ΔΔCt^.

### In vitro assays

#### Viability

Whole LLC cells (wLLC), isolated CD133 + or CD133^−^ LLC cells (2 × 10^3^), A549, murine fibroblasts, and Thp1 cell line (human monocytes) were seeded in 96-well cell culture plates and treated with 4Mu at different doses. At the indicated time points, cells were incubated with 3-[4,5-dimethylthiazol-2-yl]-2,5 diphenyltetrazolium bromide for 4 h at 37 °C with 5% C₂O₂. Isopropanol/hydrochloric acid was used to stop the reaction. Absorbance was determined at 560 nm. The experiment was carried out three times independently in 4 replicates.

Viability assays were performed incubating with 4Mu 250 µM (LLC cells) and different doses of paclitaxel (6 mg/mL) and cisplatin (1 mg/mL).

To evaluate the effect of exogenous HA, CD133 + cells (2 × 10^3^/well) were incubated with 250 µM 4Mu/ 5 nM Pa/4Mu + Pa for 72 h. After washing, they were subsequently incubated with high molecular weight (HMW) HA 200 µg/µl for other 48 h. Finally, viability was measured as described above. Recombinant HMW-HA (native HA) 1.5–1.8 × 106 Da (CPN spol. s.r.o, Czech Republic) was kindly donated by Dr Laura Alaniz.

### mRNA Expression in cell lines

mRNA levels were determined in murine LLC cells and human A549 cells with real-time qPCR when cells were exposed to 250 µM 4Mu (LLC) or 500 µM 4Mu (A549) for 24 h / 0.5 nM Pa or combined treatment . Some wells were washing and subsequently incubated with high molecular weight (HMW) HA 200 µg/µl for 48 h to determine the effect of exogenous HA.

### Clonogenic assays

For colony formation assays, 500 CD133^+^ or CD133^−^ LLC cells were treated with paclitaxel 0.5 nM or cisplatin 0.125 nM, and/or 4Mu 250 µM for 24 h. Cells were washed twice with PBS, trypsinized, plated onto 60-mm dishes, and incubated for 2 weeks before staining with crystal violet. Untreated cells were used as controls. Colonies, composed of 45–50 cells, were quantified under phase-contrast light microscopy. Three independent experiments were performed, in triplicates.

### Three-dimensional spheroid assays

Ninety-six-well tissue culture plates were coated with 75 µL of 1% agarose in PBS. CD133^+^ or CD133^−^ LLC cells were treated with paclitaxel 0.5 nM or cisplatin 0.125 nM, in combination or not with 4Mu 250 µM for 24 h. Then, cells were washed twice with PBS, trypsinized, and seeded at 5 × 10^3^ cells/well in 150 µL of 2% FBS DMEM to obtain a single homotypic spheroid per well. The spheroid size was measured on day 7 using an inverted microscope and photographed. Length and width were measured using the ImageJ program (NIH). Spheroid volume was expressed as arbitrary units.

### Soft-agar colony formation assay

For anchorage-independent cell growth, 500 CD133 + or CD133− LLC cells were treated with paclitaxel 0.5 nM and/or 4Mu 250 µM for 24 h. Cells were washed twice with PBS, trypsinized, plated onto 60-mm dishes, and incubated in soft agar according to the protocol described by Borowicz et al. for 10 days^62^. Untreated cells were used as controls. Colonies were quantified under phase-contrast light microscopy.

### Apoptosis assay

Morphological features associated with apoptosis were analyzed by acridine orange and ethidium bromide staining. LLC cells were treated with 4Mu 250 µM and/or Pa 0.5 nM for 72 h and resuspended in the dye mixture (100 μg/mL acridine orange and 100 μg/mL ethidium bromide in PBS) and visualised using fluorescence microscopy The percentage of apoptotic cells or apoptotic index was calculated as: apoptotic cells (%) = (total number of cells with apoptotic nuclei/total number of cells counted) × 100.

### Statistical analysis

All experiments were repeated at least 2 or 3 times. Values were expressed as mean ± SEM. ANOVA and multiple comparison tests evaluated the statistical differences between groups using Prism software (GraphPad, San Diego, CA, USA). A *p* value of < 0.05 was considered as significant.

## Data availability 

Data supporting reported results regarding patients can be found at https://xena.ucsc.edu/.

### Supplementary Information

Supplementary Figures.
