# Supplementary material for: Targeting hyaluronan metabolism-related molecules associated with resistant tumor-initiating cells potentiates chemotherapy efficacy in lung cancer
Source: Sci Rep. 2024 Jul 22;14:16803. doi: 10.1038/s41598-024-66914-0 (PMC11263553; doi:10.1038/s41598-024-66914-0)
Supplement: Supplementary file 1 — Supplementary Figures. [file 41598_2024_66914_MOESM1_ESM.docx]

**Supplementary Materials:**


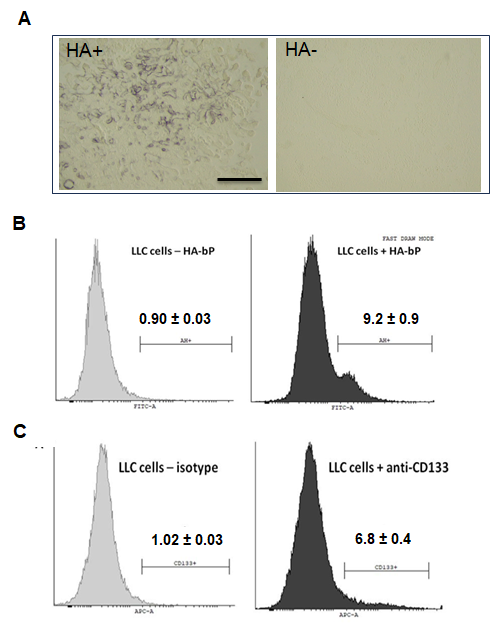


**Figure S1**: HA in murine lung cancer cells. A) Representative HA-positive staining from C57BL/6 mice lungs with LLC-established tumors through histochemistry using HAbP. HA deposits are seen in black (left). Histochemistry with HAbP in tumor samples treated with hyaluronidase showed no deposits of HA (right). Tumor region magnification (200X). Scale bar, 50 μm. B) Assessment of HA expression in the LLC cell line was performed by flow cytometry. LLC cells were incubated in the presence of biotinylated HAbP (+HAbP) or not (control; -HAbP) and then stained with avidin-FITC. Cells were fixed and subjected to flow cytometry (FACS Aria, BD). The percentage of HA+ LLC cells was analyzed using Cyflogic software. C)The percentage of CD133+ cells was determined by immuno-staining of LLC cells with anti-CD133- Allophycocyanin (APC). Cells were fixed and analyzed by flow cytometry (FACS Aria) and Cyflogic software.

**
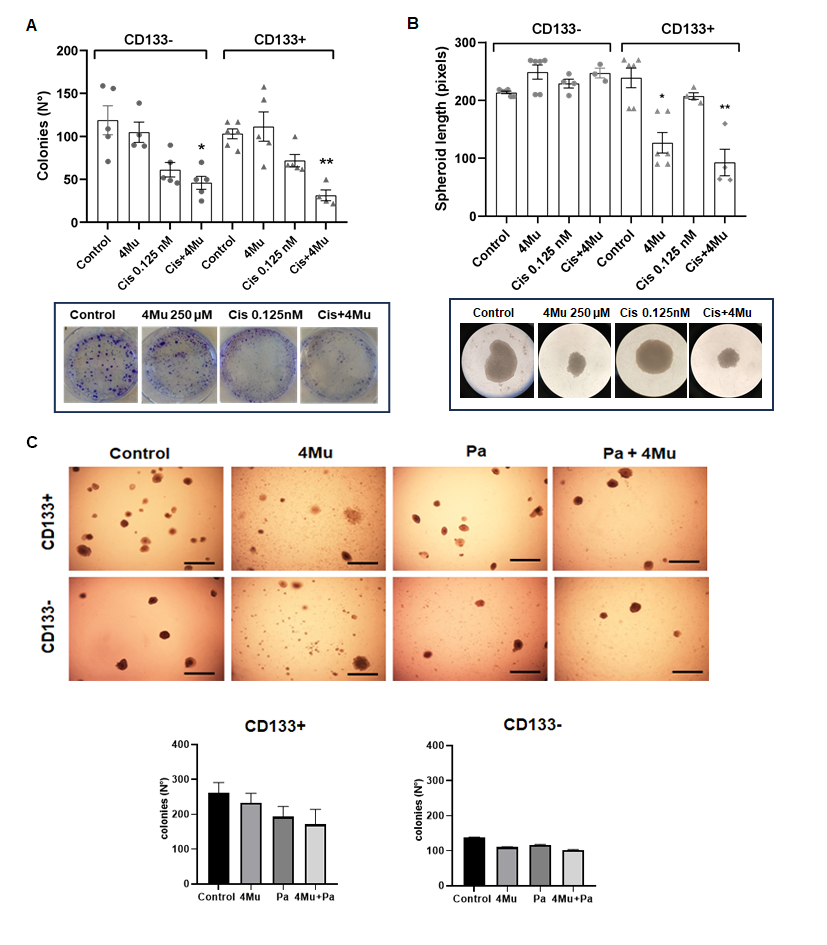
**

**Figure S2:** Effects of 4Mu plus cisplatin chemotherapy on CSC. A) Clonogenic assays were performed with CD133+ and CD133- LLC cells, treated or not with 4Mu 250 μM and/or cisplatin 0.125 nM. Briefly, cells were seeded in a 6 well plate (500 cells per well) for 15 days, fixed with methanol stained with crystal violet. Foci numbers were quantified. *p<0.05, **p<0.01 vs. Control, Kruskal-Wallis test. B) Three-dimensional spheroid assays for CD133+ and CD133- LLC cells treated or not with 4Mu 250 μM and/or cisplatin (Cis) 0.125 nM. Cells were seeded in a 96 well plate (5x10³ cells/well) coated with agarose 1% and incubated for at least 7 days, until a single homotypic spheroid per well was obtained. The spheroid diameter was measured using ImageJ software (NIH). *p<0.05, **p<0.01 vs. Control, Kruskal-Wallis test. C) Soft-agar anchorage-independent growth assay were performed with CD133+ and CD133- LLC cells, treated or not with 4Mu 250 μM and/or Pa 0.5 nM. Briefly, cells were grown in a layer of soft agar mixed with cell culture medium over another layer of soft agar, in a 6-well plate (500 cells per well). After 10 days, colony numbers were quantified with non-significant differences between groups of treatments. Kruskal-Wallis test.

**
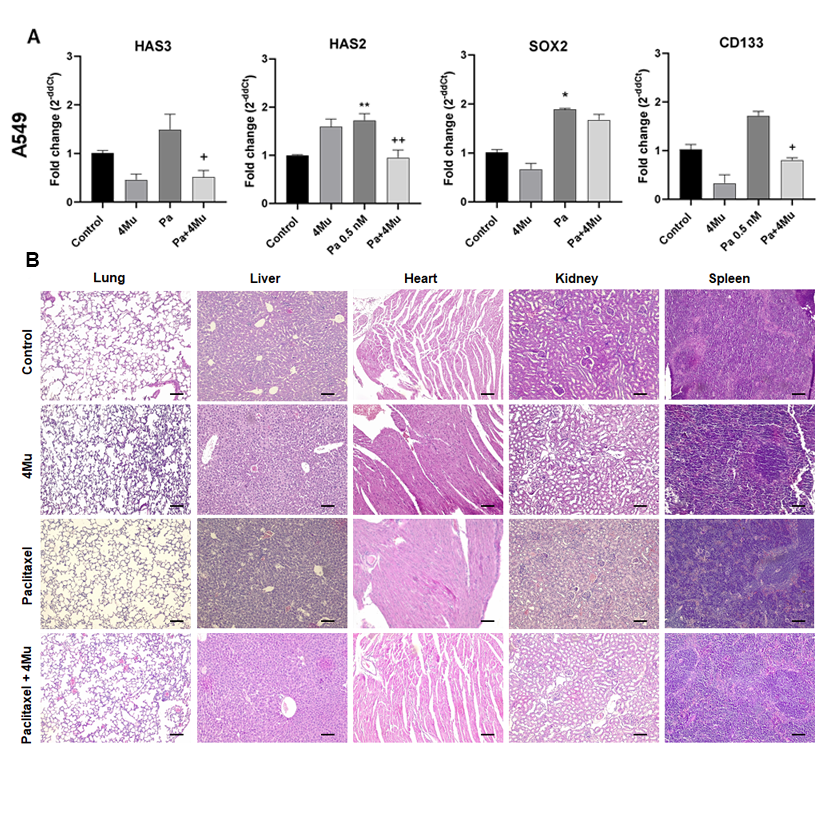
**

**Figure S3:** A) Gene expression levels of HA metabolism-associated molecules and CSC-related factors in human A549 cells. Gene expression was determined through RT-qPCR in cells treated or not with 4Mu 250 μM and/or paclitaxel (Pa) 0.5 nM for 24 hr. *p<0.05, **p<0.01 vs Control. +p<0.05, ++p<0.01 vs. Pa, Kruskal-Wallis test.

B) Representative haematoxylin-eosin stain photomicrographs of internal organs from LLC tumor-bearing C57BL/6 mice. Histology analysis of paclitaxel toxicity showed no acute tissue damage when using the dose of 10 mg/kg. Scale bar 100µm
